# Diabetes Mellitus and Depressive Disorder-Related Mortality in the United States (1999–2020): A Demographic Analysis of Long-Term Trends

**DOI:** 10.7759/cureus.92019

**Published:** 2025-09-10

**Authors:** Maira Jalil, Khushboo Rani, Lekshmi Ravi, Tshering W Sherpa, Mira S Aridas

**Affiliations:** 1 Internal Medicine, Faculty of Medicine University of Debrecen, Debrecen, HUN; 2 Internal Medicine, Goa Medical College and Hospital, Bambolim, IND; 3 Internal Medicine, North Alabama Medical Center, Florence, USA; 4 Internal Medicine, Birat Medical College, Biratnagar, NPL; 5 Neonatal and Developmental Medicine, Singapore General Hospital, Singapore, SGP

**Keywords:** age-adjusted mortality rate, cdc mcd, depressive disorder, diabetes mellitus, retrospective study

## Abstract

Introduction: Diabetes mellitus remains a significant public health challenge in the United States, with depressive disorder frequently compounding its burden. Together, these comorbid conditions not only worsen clinical outcomes but may also drive long-term mortality. However, national-level data exploring mortality trends at their intersection remain limited.

Objective: This study aims to analyze mortality trends and demographic disparities in diabetes mellitus with depressive disorder as a contributing cause using the Centers for Disease Control and Prevention (CDC) Multiple Causes of Death (MCD) database from 1999 to 2020.

Materials and methods: A retrospective observational study was conducted using the CDC MCD database to assess mortality trends in individuals aged 25 years and older in the United States from 1999 to 2020. The study included deaths where diabetes mellitus (ICD-10: E10-E14) was listed as the underlying cause and depressive disorder (ICD-10: F32) as a contributing cause. Data were analyzed by age, gender, race, geographic region, and place of death. Age-adjusted mortality rates (AAMR) and annual percentage change (APC) were calculated.

Results: A total of 13644 deaths were recorded. The AAMR for diabetes mellitus with depressive disorder showed an initial increase (6.04% APC from 1999 to 2002), but declined significantly (-3.49% APC from 2002 to 2010) and then had a significant incline (3.32% APC from 2010 to 2020). The highest mortality was observed in females (n = 7842, 57.5%), White individuals (n = 11885, 87.1%), and metropolitan areas (n = 10618, 77.8%). Temporal trends showed an increase in AAMR in males (+11.47% APC from 2018 to 2020) and White individuals (+4.04% APC from 2011 to 2020), with disparities noted across demographic and geographic factors.

Conclusions: This study highlights significant mortality trends increasing in diabetes mellitus with depressive disorder, with disparities by gender, race, and location. Findings underscore the need for targeted prevention strategies and improved healthcare access.

## Introduction

Diabetes mellitus is a chronic metabolic condition that remains a significant public health burden in the United States, with approximately 38.4 million people (11.6% of the population) affected in 2021 [1]. That year, it ranked as the eighth leading cause of death, contributing to more than 103,000 fatalities [2]. Type 2 diabetes is primarily associated with obesity, physical inactivity, family history, and specific ethnic backgrounds, whereas type 1 diabetes typically arises from autoimmune destruction of pancreatic β‑cells [3]. Beyond glycemic disturbances, diabetes markedly increases the risk of cardiovascular, renal, and neurologic complications, leading to reduced quality of life and high healthcare costs [4]. Prevalence and mortality rates are disproportionately higher among American Indian/Alaska Native adults, non‑Hispanic Black adults, and Hispanic adults, particularly in the southeastern United States [1,5].

Depression commonly occurs alongside diabetes mellitus, with each condition increasing the risk and severity of the other. People with diabetes are about twice as likely to develop depression, which can lead to poor blood sugar control and a higher risk of death [6]. Certain groups are more affected; women and older adults show higher rates, while men often remain undiagnosed [7]. Racial and ethnic minorities, particularly African American individuals and Hispanic individuals, face greater challenges due to limited healthcare access [8]. Rural communities also experience more barriers to care, underscoring the importance of improving mental health support for those managing diabetes.

Diabetes and depression share overlapping pathophysiological mechanisms, including chronic inflammation, HPA axis dysregulation, and autonomic imbalance [9]. Hyperglycemia promotes pro-inflammatory cytokines, while depression worsens glycemic control through poor self-care and lifestyle behaviors. Together, they increase the risk of complications and mortality. A meta-analysis by Park et al. found a 46% higher mortality risk in individuals with both conditions, and Zhang et al. reported depression as an independent predictor of mortality in diabetics. However, few studies explore their co-occurrence in cause-of-death data [10,11]. This study uses the Centers for Disease Control and Prevention (CDC) Multiple Causes of Death (MCD) database to examine national mortality patterns involving comorbid diabetes and depression.

This study aims to assess mortality trends in diabetes mellitus where depressive disorder is a contributing cause of death using the CDC MCD database. This study analyzes differences by sex, race, and geographic location to identify potential disparities in mortality patterns.

## Materials and methods

A retrospective original research study was conducted using the CDC Wide-Ranging Online Data for Epidemiologic Research (WONDER) MCD database [12]. The study utilized publicly available, de-identified mortality data based on U.S. death certificates for the years 1999-2020. Data extraction was performed in July 2025, and because the dataset contains de-identified, publicly accessible information, the study was classified as non-human participant research and therefore exempt from Institutional Review Board approval [13].Mortality data were retrieved for individuals aged 25 years and older, representing a population more reflective of adult-onset mortality patterns. Diabetes mellitus (ICD-10 codes E10-E14) was defined as the underlying cause of death, while depressive disorder (ICD-10 code F32) was captured as a multiple cause of death, enabling analysis of comorbid contributions. The study included demographic variables such as sex (male and female) and race (American Indian or Alaska Native individuals, Asian or Pacific Islander individuals, Black or African American individuals, and White individuals). Geographic variables were classified using the 2013 urbanization scheme, dividing areas into metropolitan (large central metro, large fringe metro, medium metro, small metro) and non-metropolitan (micropolitan and non-core) regions [14]. The place of death was also categorized into medical facilities, homes, hospices, nursing/long-term care facilities, and other settings.

Age-adjusted mortality rates (AAMR) per 1,000,000 population were calculated using the U.S. 2000 Standard Population, facilitating valid comparisons across years and subgroups [15]. Descriptive statistics, including total counts and percentage distributions, were computed to summarize demographic and geographic patterns in mortality.

To assess temporal trends, JoinPoint Regression Analysis was performed using JoinPoint Software version 5.3.0.0 (November 2024; National Cancer Institute, Bethesda, MD, USA). This statistical method estimated annual percentage changes (APC) in AAMR over the study period. Trends were further stratified by sex, race, and urbanization to identify statistically significant shifts in mortality related to diabetes and comorbid depression.

## Results

In the years 1999-2020, the CDC MCD database recorded 13,644 deaths in the United States among individuals aged 25 years and older. Among these deaths, diabetes mellitus (ICD-10: E10-14) was listed as the underlying cause of death, and depressive disorder (ICD-10: F32) was recorded as a multiple cause of death. These cases were included in the study (n = 13,644). The crude mortality rate for diabetes mellitus with depressive disorder as a contributing cause was three per 1,000,000 population. Deaths due to causes other than these criteria were excluded.

In terms of demographic characteristics, a higher proportion of deaths occurred among females (57.5%, n = 7,842) compared to males (42.5%, n = 5,802), indicating a possible gender-related disparity in mortality burden associated with diabetes mellitus and depressive disorder. Racially, White individuals accounted for the majority of deaths (87.1%, n = 11,885), followed by Black or African American individuals (10.2%, n = 1,387), Asian or Pacific Islander individuals (1.9%, n = 253), and American Indian or Alaska Native individuals (0.9%, n = 119). The mortality burden was highest among White individuals, highlighting racial disparities in mortality trends related to these comorbid conditions. Geographic analysis revealed that most deaths occurred in metropolitan areas (77.8%, n = 10,618), while non-metropolitan areas accounted for 22.2% (n = 3,026). The most significant proportions of deaths occurred in nursing homes or long-term care facilities (36.1%, n = 4,920), followed by deaths at home (35.1%, n = 4,785), medical facilities (24.0%, n = 3,273), and hospice facilities (1.0%, n = 143). These data suggest that a considerable number of individuals died outside of hospital settings, particularly in long-term care or home environments; the overall demographic characteristics of the study are included in Table 1.

**Table 1:** Demographic characteristics of the study

Demographic variable	Number of deaths (n)	Percentage (%)
Gender		
Male	5802	42.50
Female	7842	57.50
Race		
American Indian or Alaska Native	119	0.90
Asian or Pacific Islander	253	1.90
Black or African American	1387	10.20
White	11885	87.10
Urbanization		
Metropolitan area	10618	77.80
Large central metro	3136	23.00
Large fringe metro	2781	20.40
Medium metro	3131	22.90
Small metro	1570	11.50
Non-metropolitan area	3026	22.20
Micropolitan	1716	12.60
Non-core	1310	9.60
Place of death		
Medical facility	3273	24.00
Decedent's home	4785	35.10
Hospice facility	143	1.00
Nursing home/long-term care	4920	36.10
Other	523	3.90

**Figure 1:**
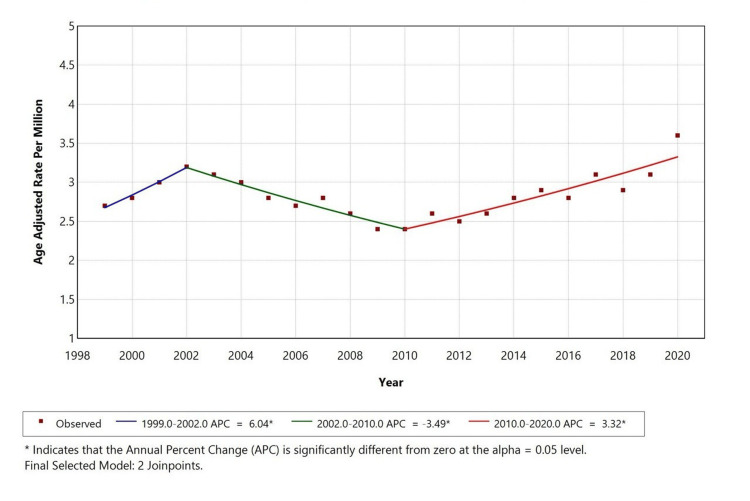
Overall AAMR among adults aged 25+ in the United States (1999-2020) AAMR: age-adjusted mortality rates, APC: annual percentage change

Over the study period, the AAMR for diabetes mellitus with depressive disorder as a contributing cause showed a fluctuating pattern (Figure 1). From 1999 to 2002, there was an increase in AAMR with an APC of 6.04 (p = 0.05), followed by a significant decline from 2002 to 2010 (APC = -3.49, p = 0.05) and then a significant rise in APC to 3.32 during the last decade. Significant inflection points were observed in 1999 and 2010, indicating treatment advances, changes in disease prevalence, and public health interventions.

**Figure 2:**
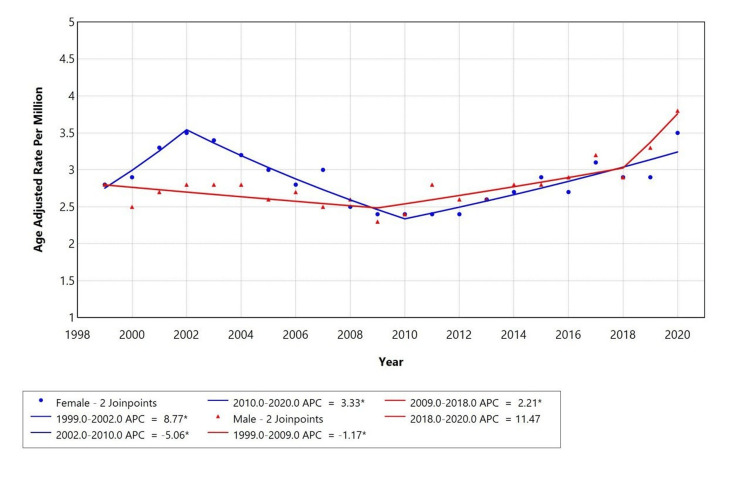
Trends in sex-stratified AAMR among adults aged 25+ in the United States (1999-2020) AAMR: age-adjusted mortality rates, APC: annual percentage change

Gender-specific trends revealed differences in trajectory (Figure 2). Females experienced an initial increase from 1999 to 2002 (APC = 8.77), a decline from 2002 to 2010 (APC = -5.06), and a subsequent rise through 2020 (APC = 3.33). In contrast, males showed a decline from 1999 to 2009 (APC = -1.17), followed by increases from 2009 to 2018 (APC = 2.21) and 2018 to 2020 (APC = 11.47), though the latter was not statistically significant.

**Figure 3:**
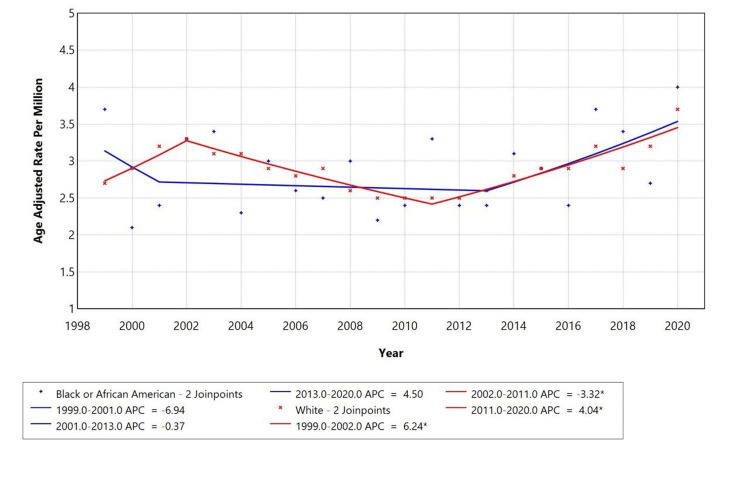
Trends in AAMR stratified by race among adults aged 25+ years in the United States (1999-2020) AAMR: age-adjusted mortality rates, APC: annual percentage change

Racial disparities were observed in mortality trends (Figure 3). Black or African American individuals had the highest AAMR, with a non-significant increase in APC (4.5) from 2013 to 2020. White individuals experienced a significant rise in mortality from 2011 to 2020 (APC = 4.04, p = 0.05). Trends for American Indian/Alaska Native and Asian or Pacific Islander individuals were not displayed due to data suppression for counts <10, limiting reliable trend analysis.

## Discussion

A retrospective study was conducted using the CDC MCD database to assess mortality trends of diabetes mellitus (ICD E10-E14) with depressive disorders (ICD F32) among adults aged ≥25 years in the United States from 1999 to 2020. This study revealed 13,644 total deaths where diabetes was the underlying cause and depression a contributing factor. Over the study period, the AAMR for diabetes mellitus with depression initially increased from 1999 to 2002 (APC: +6.04%), followed by a decline from 2002 to 2010 (APC: -3.45%), and then continued to rise again from 2010 to 2020 (APC: +3.32%). The highest mortality was observed in females (57.5%), White individuals (87.1%), and metropolitan regions (77.8%).

The relationship between diabetes and depression is complex and bidirectional. Depression is associated with increased risk of developing type 2 diabetes due to behavioral (e.g., physical inactivity, poor diet) as well as physiological mechanisms (e.g., HPA axis dysregulation, inflammation) [16,17]. Conversely, individuals with diabetes are more likely to develop depression due to disease burden, complications, and lifestyle limitations [18]. Depression in diabetic patients is linked with poor glycemic control, reduced adherence to medications, and a higher risk of macrovascular and microvascular complications [8,19]. These factors, particularly when coexisting over time, contribute significantly to elevated morbidity and mortality risk. The compounded stress of managing both conditions, combined with possible underdiagnosis of depression in medical settings, likely accelerates adverse outcomes [20].

This study found that diabetes with comorbid depression had an overall AAMR of 3 per million, with an APC that suggests a statistically significant change over time. Previous literature has consistently shown elevated mortality risk among patients with both conditions. For instance, Pan et al. reported a 1.5-fold increased mortality risk in individuals with both diabetes and depression [21], while Zhang et al. showed that comorbid depression increased the risk of all-cause mortality by 52% in diabetic adults [11]. Our findings align with this pattern, though the crude mortality rate appears modest, possibly due to the strict coding criteria or underreporting of depression on death certificates. Improvements in diabetes care, including the use of medications and education, may also partially explain the moderated trend observed in recent years [22].

Females accounted for 57.5% of total deaths, likely reflecting the higher lifetime prevalence of depression among women and greater likelihood of symptom reporting and healthcare utilization [23]. However, under-recognition of depression in women and barriers to accessing appropriate care (especially among older adults) may contribute to greater late-stage mortality [24]. Although males had fewer deaths overall, their mortality increased modestly from 2009 to 2018, followed by a sharp but statistically insignificant rise from 2018 to 2020. This pattern may still reflect emerging risks due to delayed diagnosis and lower engagement with mental health services. White individuals accounted for 87.1% of deaths, appearing overrepresented relative to their population share. Black or African American individuals accounted for 10.2%, slightly underrepresented, which may reflect underdiagnosis or misclassification of depression rather than improved outcomes. Studies show Black patients with diabetes and depression face reduced access to care, lower treatment rates, and higher complication risks [25,26]. These disparities are further influenced by structural inequities such as economic hardship and systemic healthcare barriers [27].

Geographic disparities were significant. Most deaths occurred in metropolitan areas (77.8%). This may reflect population distribution and greater access to diagnostic services. However, non-metropolitan areas still accounted for over 22% of deaths, and previous studies have shown poorer outcomes in rural areas, linked to limited mental health infrastructure, provider shortages, and definitional challenges that complicate rural health policy [28]. Lifestyle risk factors such as obesity, smoking, and sedentary behavior may be more prevalent in underserved rural communities, compounding risk [29].

Temporal trends revealed that AAMR varied over the study period, with statistically significant changes seen across sex and race categories. Prior research also shows dynamic shifts in diabetes-related mortality, influenced by evolving treatments, early diagnosis, and health policy interventions [30,31]. However, trends in comorbid depression remain less well studied. The stable-to-rising trends seen in our study suggest that comorbid mental health conditions may not be adequately addressed in chronic disease management.

These findings underscore the need for integrated care models that treat both diabetes and mental health concurrently. Future studies should investigate disparities in mental health screening, interventions for high-risk racial and geographic groups, and policy changes to improve access to behavioral healthcare. Efforts to reduce preventable mortality must focus on coordinated chronic disease and mental health care delivery, particularly in underserved populations.

Limitations

This study has limitations, including potential coding inaccuracies and misclassification in death certificate data. The lack of information on comorbidities and treatment history restricts the clinical context. Temporal changes in diagnostic and reporting practices may have influenced mortality trends. As a retrospective observational study, causality cannot be inferred. Additionally, confounders such as socioeconomic status, healthcare access, and lifestyle factors were not included in the MCD database, which may affect interpretation.

## Conclusions

This study highlights the evolving burden of mortality trends in patients with diabetes mellitus and depressive disorder. Between 1999 and 2020, age-adjusted mortality trends exhibited dynamic shifts with an overall increase over the recent years. There were disparities observed across gender, race, and geography, with higher mortality observed in females, White individuals, and metropolitan populations. These findings emphasize the complex relationship between chronic physical and mental health conditions and the urgent need for holistic integrated care approaches. Depression remains largely underdiagnosed and undertreated in individuals with diabetes, leading to a compounded risk of adverse outcomes. Targeted public health strategies are essential to address these disparities, improve access to mental health services, and reduce mortality. Future studies and research should explore causal pathways, identify gaps in care in high-risk populations, and evaluate the effect of timely interventions that bridge physical and mental health.
